# From the Intensive Care Unit to Recovery: Managing Post-intensive Care Syndrome in Critically Ill Patients

**DOI:** 10.7759/cureus.61443

**Published:** 2024-05-31

**Authors:** Mfonido Ekong, Tejbir Singh Monga, Jean Carlo Daher, Mutyala Sashank, Setareh Reza Soltani, Nkiruka Lauretta Nwangene, Cara Mohammed, Fellipe Feijo Halfeld, Leen AlShelh, Fernanda Ayumi Fukuya, Manju Rai

**Affiliations:** 1 Internal Medicine, St. George's University School of Medicine, True Blue, GRD; 2 Internal Medicine, Spartan Health Science University, Vieux Fort, LCA; 3 Medicine, Lakeland Regional Health, Lakeland, USA; 4 Medicine, Universidad de Ciencias Medicas Andres Vesalio Guzman, San Jose, CRI; 5 Internal Medicine, Davao Medical School Foundation Inc., Davao, PHL; 6 Advanced Diagnostic & Interventional Radiology Center (ADIR), Tehran University of Medical Sciences, Tehran, IRN; 7 Internal Medicine, Campbell University, Buies Creek, USA; 8 Orthopaedic Surgery, Sangre Grande Hospital, Sangre Grande, TTO; 9 Internal Medicine, Federal University of Rio de Janeiro, Rio de Janeiro, BRA; 10 Internal Medicine, Medical University of Lublin, Lublin, POL; 11 Internal Medicine, University of Mogi das Cruzes (UMC), Sao Paulo, BRA; 12 Biotechnology, Shri Venkateshwara University, Punjab, IND

**Keywords:** multidisciplinary approach, quality of life, mental health, cognitive impairment, physical therapy, assessment scales, screening tools, rehabilitation, critically ill patients, post-intensive care syndrome (pics)

## Abstract

Post-intensive care syndrome (PICS) is the term used to describe the decline in the physical, cognitive, and/or mental condition of individuals who have been discharged from the intensive care unit (ICU). This complication could result in a significant reduction in quality of life, with some patients experiencing symptoms of prolonged weakness, depression, anxiety, and post-traumatic stress disorder (PTSD). Intensive care advancement over the years has resulted in an increase in ICU survival rates and a proportional increase in PICS, creating a need for more in-depth research into the prevention and management of the disease. Hence, this study aims to examine the present body of literature on PICS, encompassing its underlying physiological processes and elements that contribute to its development, methods for evaluating and diagnosing the condition, current treatment choices as well as potential new approaches, and the constraints in managing PICS and the necessity for further investigation. In this article, studies were compiled from several databases, including, but not limited to, Google Scholar, PubMed, and Cochrane Library. These studies were reviewed, and their data were used to highlight important aspects regarding the efficacy of current PICS screening tools, the optimization and limitations of both pharmacologic and non-pharmacologic treatment methods, and the feasibility and safety of emerging treatments and technologies. The major conclusions of this review were centered around the need for multidisciplinary management of PICS. From pharmacological management using analgesia to non-pharmacological management using early mobilization and exercise therapy, the effective treatment of PICS requires a multifaceted approach. Patient follow-up and its importance were touched upon, including strategies and policies to bolster proper follow-up, thereby increasing favorable outcomes. Lastly, the importance of family involvement and the increased need for research into this topic were highlighted.

## Introduction and background

Post-intensive care syndrome (PICS) is a notable issue in critical care medicine that affects individuals who have survived a critical illness. It presents considerable challenges for patients, their families, and healthcare systems. PICS refers to the continued presence or worsening of physical, cognitive, or mental health deficits after a critical illness, lasting beyond the initial period of medical treatment [1-3]. The syndrome comprises a diverse range of deficits, such as muscular weakness, cognitive dysfunction, depression, anxiety, and post-traumatic stress disorder (PTSD) [1].

The occurrence of PICS varies based on the time elapsed after discharge and the particular attributes of the patient population [4]. Research has shown that a significant percentage, ranging from 50% to 80%, of patients who are released from the intensive care unit (ICU) experience a variety of challenges that continue long after they leave the hospital. A considerable percentage, ranging from 30% to 80%, of persons who survive stays in the ICU experience cognitive impairment. This impairment can vary in terms of severity and length, and in some cases, it can remain for several years. Simultaneously, psychological disorders such as anxiety, sadness, and PTSD occur in 8%-57% of patients, and they also display enduring effects [5]. In addition, a range of new physical restrictions arise in 25%-80% of patients, accompanied by symptoms such as difficulty breathing, pain, sexual dysfunction, diminished lung function, and decreased ability to engage in physical activity [5]. The precise occurrence rate of post-ICU sequelae remains uncertain and varies considerably across different groups of patients, with certain individuals recovering without considerable damage, while others encounter problems in numerous areas [3,5].

With the evolving dynamics of critical care, it is crucial to recognize and tackle the lasting effects of ICU stays in order to enhance patient outcomes and optimize healthcare provision. Patients who have survived a severe illness frequently encounter difficulties in resuming their regular daily routines, reintegrating into the workforce, and sustaining social connections as a result of the persistent consequences of PICS [1]. Furthermore, the financial strain linked to extended hospital stays and rehabilitation emphasizes the significance of addressing PICS to maximize the allocation of healthcare resources [1]. Therefore, PICS is acknowledged as an expanding worldwide healthcare issue, and understanding its significance is crucial for providing comprehensive treatment to ICU survivors and reducing the impact of long-term illness.

Various therapies are being implemented to reduce PICS in ICUs worldwide, including rehabilitation programs and psychological assistance [6,7]. The goal is to empower patients to regain autonomy and live a normal life. Addressing PICS can reduce the economic burden on healthcare systems, leading to more sustainable systems. Effective management of PICS not only improves quality of life and reduces healthcare expenses but also enhances long-term functional condition [8]. Exercise therapy is a highly effective preventive treatment for PICS and post-ICU outcomes. Targeting modifiable risk factors and implementing lifestyle changes can enhance patients' physical resilience and overall well-being [9,10]. Therefore, a comprehensive approach is needed to help patients return to their initial state, improve their quality of life, reduce healthcare costs, and foster long-term physical autonomy post-ICU discharge.

The objective of this narrative review is to consolidate and analyze existing clinical and randomized controlled trials on PICS to provide a comprehensive overview of the current understanding of its assessment, diagnosis, management approaches, challenges, and future directions. By doing so, the review aims to identify gaps in knowledge and areas where further research is needed to improve patient outcomes and quality of life following intensive care discharge.

## Review

Methods

**Figure 1:**
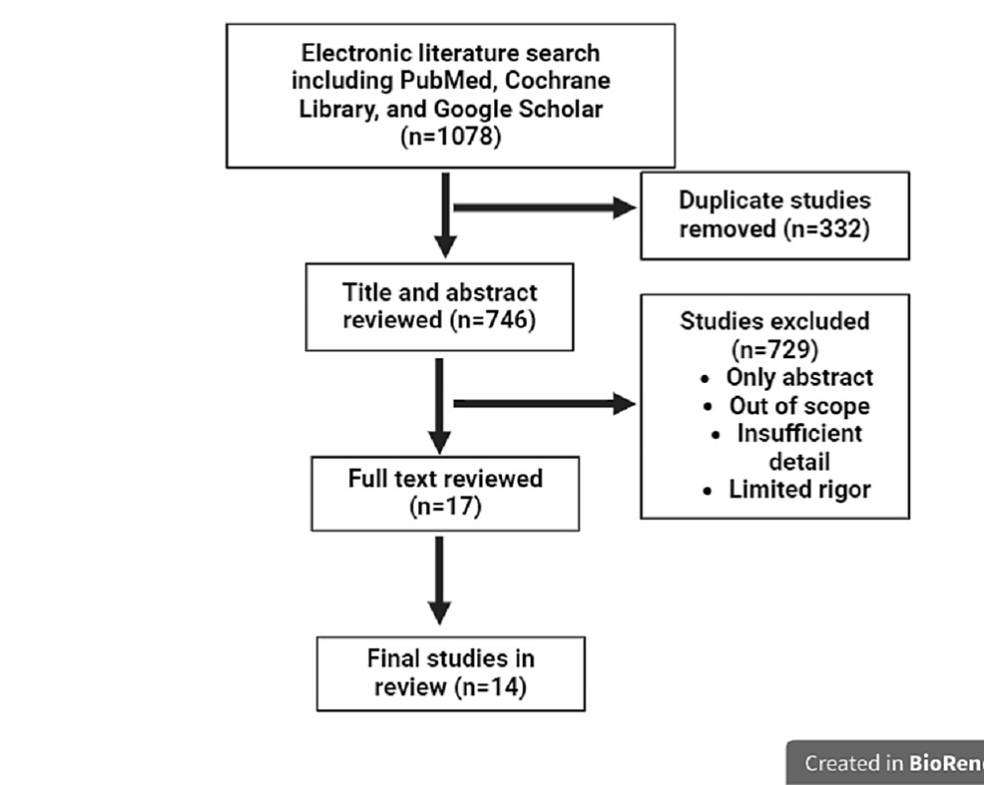
Flow diagram of the study selection for review.

This narrative review used Medline, Cochrane Library, and Google Scholar databases to search all studies, through March 2024, that reported risk factors for anxiety, depression, and PTSD; diagnostic criteria and tools; and pharmacological and non-pharmacological interventions. Of 1,078 identified studies, 14 studies comprising prospective cohorts and randomized clinical trials were included (Figure 1).

Pathophysiology of PICS

**Figure 2:**
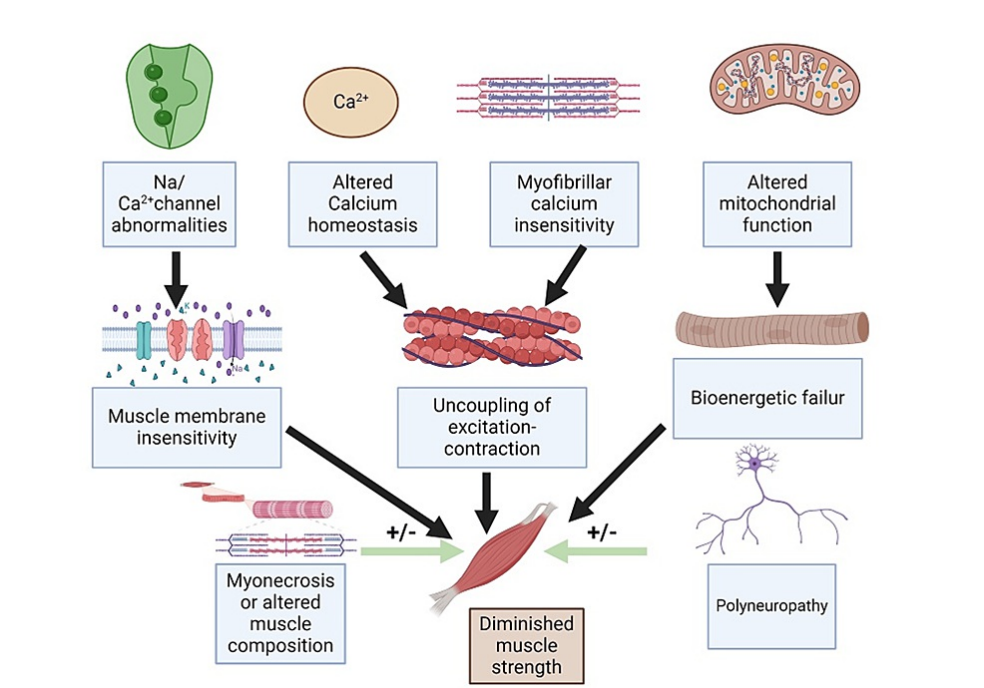
Proposed mechanism of intensive care unit-acquired weakness. Image credits: Tejbir Singh Monga

The greatest concern lies in the long-term impairment of critically ill patients, as their survival rates continue to increase. Post-ICU-acquired weakness is a condition characterized by abrupt muscle weakness in the extremities, occurring symmetrically, and accompanied by polyneuropathy [11]. This condition is associated with longer hospital stays and higher mortality rates. The observed weakness is associated with damage to small blood vessels, resulting in reduced blood supply to the nerves. This leads to muscle deconditioning and disruption of mitochondrial function, which in turn causes injury to satellite cells. Additionally, the loss of a protein called titin, as discovered in the urine, is likely responsible for muscle weakness [12,13]. However, we now lack definitive evidence regarding the specific etiology of muscle weakness, which consequently hinders the development of treatment based on solid scientific evidence [12]. The presence of persistent inflammation after ICU treatment was mostly attributed to ongoing tissue damage (Figure 2). The main reason for B-cell depletion is an increase in apoptosis and an increase in T-cell regulatory type. This leads to an inadequate adaptive response of the immune system to antigens, resulting in a higher risk of nosocomial infections [2].

PICS is also associated with cognitive deficits, mostly including anxiety, depression, and PTSD [11]. Survivors of critical illness experience a significant level of physical discomfort, which is linked to a higher likelihood of depression and anxiety in these individuals [14]. Admission to the ICU is frequently abrupt and poses a significant risk to the patient's life. Moreover, patients commonly experience post-traumatic stress, which can potentially progress to PTSD. These patients frequently report experiencing nightmares, discomfort, respiratory distress, or a sensation of suffocation while in the ICU [15]. Patients who are severely unwell after being in critical care can suffer from varying degrees of cognitive impairment, which can last for months or even years after leaving the hospital. These symptoms, including hypo-/hyperglycemia, delirium, and acute stress, were observed during the patient's hospitalization [11].

Risk factors for developing PICS

**Figure 3:**
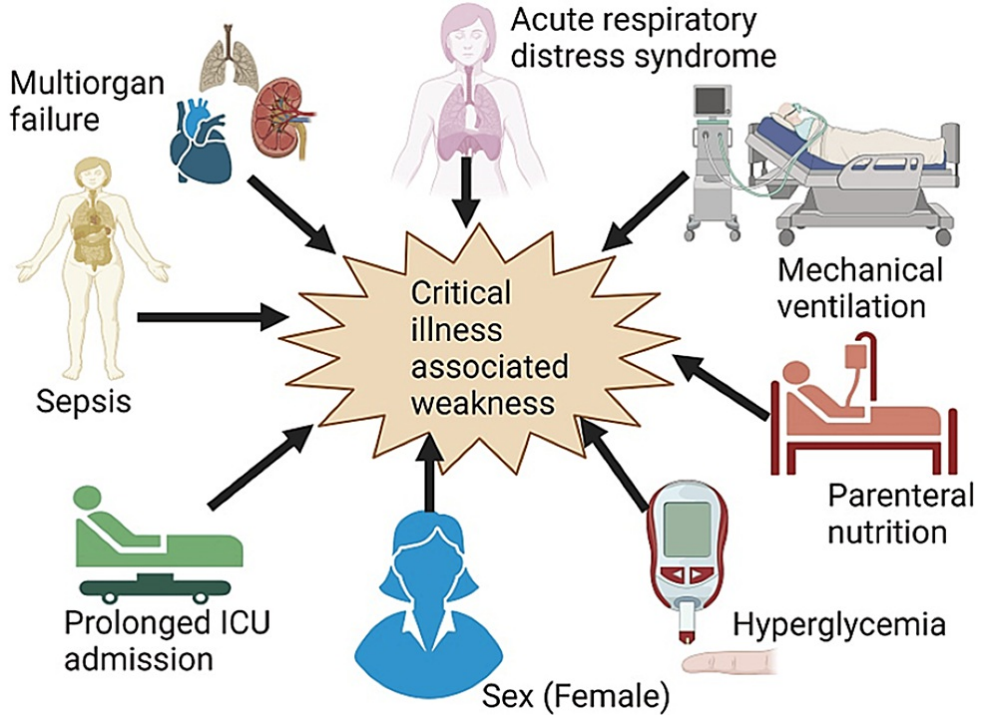
Common risk factors associated with the development of critical illness-associated weakness. Image Credits: Cara Mohammed

The degree of disease severity is a crucial aspect in determining the risk factors for PICS. Severely unwell individuals are prone to encountering difficulties such as physical, cognitive, and behavioral health issues after being discharged from the hospital [16]. Acute respiratory distress syndrome, extended mechanical ventilation, parenteral nutrition, hyperglycemia, female sex, prolonged ICU admission, and sepsis are the major risk factors associated with critical illness-associated weakness (Figure 3). Additionally, individuals with pre-existing delirium and comorbidities such as premorbid psychiatric disease, use of sedatives and analgesics, low cognitive reserve, and persistent inflammatory response syndrome are more likely to develop critical-illness-associated weakness and PICS compared to the general population [3,17]. Meanwhile, there are other risk factors for PICS, including 33 personal characteristics and 27 factors related to the ICU, which are critical for identifying individuals who are at a greater risk [16]. Elderly individuals and women are more susceptible to enduring long-term impairments in physical, cognitive, and mental well-being following a severe illness [16,18].

It has been reported that prolonged mechanical ventilation for more than seven days is a significant risk factor for PICS [3,16]. This is especially true when it leads to physical impairment and acquired neuromuscular weakness [19], which occurs in over 25% of ICU survivors [3]. The consequences of this weakness include poor mobility, recurrent falls, and quadri- or tetraparesis. Survivors who were mechanically ventilated at an older age have a 30% higher likelihood of experiencing activity of daily living (ADL) impairment [17]. Several studies have found that administering sedatives (insufficient practices and extended periods of bed-induced deep drowsiness) and analgesics can heighten the likelihood of cognitive impairment and mental health problems such as delirium [3,16,17].

Delirium is the predominant consequence associated with cognitive impairment in patients with PICS, according to multiple studies examining risk variables. The negative experiences of ICU patients, including instances of poor patient experience and delirium, significantly contribute to the development of anxiety, sadness, and PTSD [14-16,18,19]. Ahmad et al. further stated that delirium is identified as a separate risk factor that contributes to cognitive impairment after six and 12 months [17]. However, insufficient data exist to establish a strong correlation between older patients and increased vulnerability to cognitive PICS compared to younger ones. Therefore, PICS is a complex disorder that has enduring consequences for patients and their families, and gaining knowledge about the factors that increase the likelihood of developing PICS and adopting substantial interventions can be beneficial in reducing its severity.

Assessment and diagnosis of PICS

Screening Tools and Assessment Scales

Screening tools and assessment scales play a vital role in identifying and quantifying the extent of PICS-related impairments, allowing healthcare professionals to tailor interventions accordingly. These tools help in systematically assessing various domains affected by PICS, including physical function, cognition, mental health, and quality of life.

The ICU Liberation (ABCDEF) Bundle is implemented to mitigate prevalent complications observed in patients following ICU admission, encompassing pain, mechanical ventilation, sedation, delirium, mobility, and family participation [20]. A research investigation was carried out in Michigan to ascertain the variables that influenced the execution of the ICU Liberation package in 72 ICUs [21]. The study determined that the ICU Liberation Bundle exhibits a high level of efficacy in cases when there is a robust safety culture and adequate communication among staff members, leading to notable outcomes. Amidst the COVID-19 pandemic in 2019, various modifications were implemented to enhance the outcome of the ICU Liberation Bundle. One of these changes involved granting pediatric ICUs (PICUs) the authority to care for adult patients, despite their typical focus on managing metabolic and pathologic conditions specific to children [22]. Analyzing the obstacles and challenges that influenced the result facilitated the implementation of ICU emancipation and underscored the utilization of a patient-centered approach in conducting philosophical research [23,24]. Remarkably, the successful implementation of the ICU Liberation resulted in a decrease in the amount of time patients needed mechanical breathing and the length of their stay in the ICU. It also helped prevent lengthy stays in the ICU, even though not all aspects of the bundle were done equally. Although implementing all components of the Bundle resulted in a significant decrease in post-ICU morbidity, some patients did not receive appropriate treatment for the first component due to their lower pain levels compared to most ICU patients. Therefore, ensuring that staff members conduct thorough pain assessments can facilitate the implementation of all components of the Bundle [19].

At present, there is a lack of specific instruments employed for all ICU patients before they are discharged in order to detect any potential future health complications. It has been shown that certain screening methods employed during discharge have accurately assessed the patient's present condition, aiding in the identification and prevention of PICS [17]. To study the impact of ARDS after ICU admission, it is crucial to thoroughly document the patient's symptoms and test results in order to minimize the possibility of developing PICS [25]. The six-minute walk test (6MWT) is an assessment tool that plays a crucial role in measuring exercise capacity and identifying potential triggers [26]. It has shown promising results in the field of critical care physical assessment, together with the critical care physical assessment instrument (CPAx). Though lengthier stays in ICUs have been associated with PICS and post-admission morbidities, there have been cases where patients admitted for only one to two days to the ICU also experienced post-admission morbidities [27]. Weissman et al. utilized natural language processing techniques to evaluate the recording of critical disease characteristics in discharge records [25]. In addition, ICU diary programs are used to reduce psychological morbidity related to critical illness. Thus, thorough and accurate research is necessary to contribute to the creation and improvement of evaluation tools that aim to enhance outcomes for patients transferring from the ICU to recovery.

Assessments have a vital role in detecting any aberrant cognitive function. A recent study has discovered that often utilized tools for this purpose include the Hospital Anxiety and Depression Scale (HADS), Impact of Events Scale-Revised (IES-R), and the six-item Impact of Event Scale-6 (IES-6) [17]. However, by comparing all of the PICS assessment methods, it has been shown that the Montreal Cognitive Assessment (MoCA) achieved the highest score in terms of reliability and validity for assessing the cognitive function of patients after ICU admission [28]. A protocol was developed for a multicenter randomized controlled trial that aims to evaluate the effects of a post-ICU multidisciplinary follow-up on the quality of life [29]. This study highlights the increasing recognition of the importance of comprehensive post-ICU care interventions in addressing the various challenges associated with PICS, such as cognitive impairment. In addition, a comprehensive analysis and synthesis of multiple studies was performed to assess the impact of inhaled anesthetics on cognitive function in critically ill people [30]. This study emphasizes the significance of taking into account sedation methods in order to reduce cognitive loss associated with PICS.

Diagnostic Criteria

PICS is distinguished by a blend of physical, cognitive, and mental health deficiencies that might endure for an extended period even after the acute sickness has subsided. These deficits can have a substantial effect on the quality of life and functional outcomes of patients [17]. In order to efficiently handle PICS, it is crucial to have a comprehensive understanding of the diagnostic criteria for prevalent psychological illnesses that may occur in this group, such as PTSD, depression, and anxiety disorders, and objective assessments of physical functioning.

PTSD frequently occurs as a psychological outcome of critical illness and the time spent in the ICU. The Statistical Manual of Mental Disorders, Fifth Edition (DSM-5), offers precise criteria for diagnosing PTSD [31-33]. The DSM-5 outlines the diagnostic criteria for PTSD, which consist of experiencing a traumatic incident and exhibiting symptoms in four categories: intrusive symptoms, avoidance behaviors, negative changes in mood and thinking, and modifications in responsiveness and arousal. In order to meet the criteria, patients must experience the specified symptoms for a minimum of one month, and these symptoms must result in severe distress or impairment in their daily functioning. It is also crucial that individuals display at least one symptom of avoidance. Similarly, individuals who have experienced severe sickness and had treatment in the ICU are more likely to acquire PTSD as a result of the traumatic nature of their disease and treatment experience [34]. Screening ICU survivors for signs of PTSD is crucial to identify individuals who could potentially benefit from therapies such as cognitive-behavioral therapy [35]. Therefore, by applying interventions that adhere to the DSM-5 recommendations for diagnosing PTSD, healthcare practitioners can effectively recognize and treat PTSD symptoms in critically ill patients with PICS [36].

The diagnostic criteria for depression commonly encompass enduring emotions of melancholy, hopelessness, or diminished interest in formerly pleasurable activities, alterations in eating or sleep habits, exhaustion, and challenges with concentration. To be diagnosed with depression, an individual must regularly experience symptoms throughout the day for at least two weeks. One of these symptoms must be either a persistent feeling of sadness or a decreased interest in things that were once enjoyable [14]. Individuals diagnosed with PICS may encounter elevated degrees of depression as a result of the intense strain caused by their medical condition and the difficulties associated with recuperation. Prompt identification and timely intervention are essential for enhancing patient outcomes, promoting general wellness, and anticipating psychological complications following critical illness [37]. In addition, the diagnostic criteria for anxiety disorders comprise symptoms such as heightened and persistent worry or anxiety, restlessness, exhaustion, impaired concentration, irritability, muscle tension, and disruptions in sleep patterns. Generalized anxiety disorder (GAD) is characterized by persistent feelings of apprehension or anxiety that might interfere with daily activities. GAD is distinct from occasional anxieties or stress-related worry because patients with GAD routinely experience anxiety for prolonged periods, perhaps lasting for months or even years [38]. Similarly, Goldstein-Piekarski et al. conducted a comprehensive analysis that examined the co-occurrence of anxiety disorders and the influence of various exclusion criteria on the study of clinical outcomes in anxiety disorders [39]. The review highlighted the need to have a thorough understanding of the coexistence of anxiety disorders in order to enhance the accuracy of diagnosis and improve treatment results in patients with PICS.

Furthermore, individuals with PICS may have substantial limitations in their physical abilities after their time in the ICU. Quantifiable indicators such as muscle strength, endurance, mobility, and ability to do everyday tasks can offer vital information about a patient's physical recovery progress. Aglawe et al. conducted a study that investigated the physical function of critically sick patients throughout their stay in the ICU and hospital [40]. One of the procedures being examined in this study was the Physical Function ICU Test (PFIT). The assessment quantifies the strength of shoulder flexion and knee extension on a scale ranging from 0 to 5. It also examines the level of help required during sit-to-stand activity and measures step cadence in terms of the number of steps per minute. Patients may utilize assistive equipment as necessary during the examination. The test concludes if the patient is unable to lift their feet off the ground for six consecutive steps or if they cease marching for longer than two seconds. Patients who are able to march continuously for more than three minutes are given a score of 3. The study emphasized the significance of prompt evaluation and rehabilitation therapies to enhance physical functioning outcomes in critically sick patients with PICS. Customized physical therapy interventions can effectively boost functional outcomes and improve the quality of life for people diagnosed with PICS [41].

Multidisciplinary management approaches

Pharmacological Interventions

The pharmaceutical management of PICS and critically ill patients is complex and involves a combination of suitable sedation, analgesia, and addressing mental disorders while aiding in rehabilitation [42]. Given the obvious correlation between poor management and higher death rates, it is crucial to thoroughly examine research in order to encourage the appropriate utilization of available medications [43].

Most of the studies on analgesia and sedation have primarily concentrated on the management of patients in ICUs. These studies have utilized the 2018 "Clinical Practice Guidelines for Prevention and Management of Pain, Agitation/Sedation, Delirium, Immobility and Sleep Disruption (PADS) in the ICU". These guidelines recommend the utilization of pain assessment scales such as Behavioral Pain Scale (BPS) and Critical-Care Pain Observation Tool (CPOT), as well as the use of the Richmond agitation-sedation scale (RASS) for sedation assessment, in order to adjust medication dosages. Propofol and benzodiazepines are the most commonly utilized medications in the context of sedatives [43]. The objective is to provide mild sedation by employing multimodal methods, such as ensuring sufficient pain relief and facilitating proper sleep [44]. Both substances can induce withdrawal symptoms if used for longer than one week. However, propofol IV injections have demonstrated efficacy due to their rapid onset and consistent pharmacokinetic properties, which are unaffected by renal and hepatic failure in patients. The adverse effects include bradycardia, injection site tenderness, respiratory depression, and systemic vasodilation, which may result in hypotension. The incidence of the propofol-infused condition, albeit rare, is associated with a death rate of 33%; however, it only represents 1% of the total cases [42]. Examining the choice among benzodiazepines, such as midazolam, lorazepam, and diazepam: midazolam exhibits a quick onset time, but it has a tendency to build up in those who are obese or have renal failure. Despite this, it is frequently utilized. Lorazepam, on the other hand, has a slower beginning of action but has fewer interactions and is easier to modify and monitor. Meanwhile, diazepam has a rapid onset and can be maintained with repeated administrations [42]. Moreover, patients who were given benzodiazepines were more likely to develop delirium. Therefore, while considering both choices, it was recommended to use propofol instead of midazolam [42]. Another alternative that is less risky is clonidine, which might also be more economical when compared to benzodiazepines. Typically, many medications are required to maintain the desired level of sedation [43].

Effective pain management is crucial and can lead to a decrease in the required dosage of sedatives when administered appropriately. Individual assessment is necessary to determine the optimal targets for pain treatment [43]. IV opioids, NSAIDs, and anticonvulsants are commonly used for pain management. However, it is important to take into account the potential adverse effects before deciding on the appropriate analgesic treatment. The use of opioids in combination with gabapentin, carbamazepine, and pregabalin is strongly suggested for treating neuropathic pain. However, it is essential to closely monitor the use of opioids due to their potential side effects, including low blood pressure, decreased breathing, and impaired bowel movement [42].

Brain impairment in critically ill individuals can result directly from the iatrogenic effects of opioids and benzodiazepines [34]. Hence, it is crucial to utilize it in an appropriate way. Patients with PICS commonly experience a high occurrence of depression, anxiety, PTSD, and sleep difficulties, which result in long-term disability [41]. Ketamine has been regarded as an alternate antipsychotic and sedative drug for treating PTSD, especially in patients with asthma and hypotension [45]. Quetiapine and risperidone, both classified as atypical antipsychotic medicines, are commonly prescribed in therapeutic settings. In contrast, benzodiazepines are not favored due to their elevated potential for addiction [46]. Delirium is a frequently occurring disorder, and the use of drugs such as haloperidol may be used to control hyperactive behavior and symptoms associated with stress or delirium [47]. Prazosin showed efficacy in the treatment of nightmares and insomnia [46]. In addition to its sedative and analgesic qualities, dexmedetomidine was found to be effective in inducing a regular sleep pattern [41].

It is widely recognized that frailty in the ICU increases the likelihood of death due to its immediate and long-term negative effects. Thus, rehabilitation plays a crucial role in the process of recovery. There is no conclusive evidence about the efficacy of drugs such as oxandrolone and anabolic steroids, such as testosterone, in enhancing muscle growth, accelerating recovery, and facilitating weight gain [48]. However, research conducted on 48 patients who had thermal injuries and were administered oxandrolone and anabolic steroids demonstrated accelerated weight growth and improved healing rates [49].

Non-pharmacological Interventions

**Figure 4:**
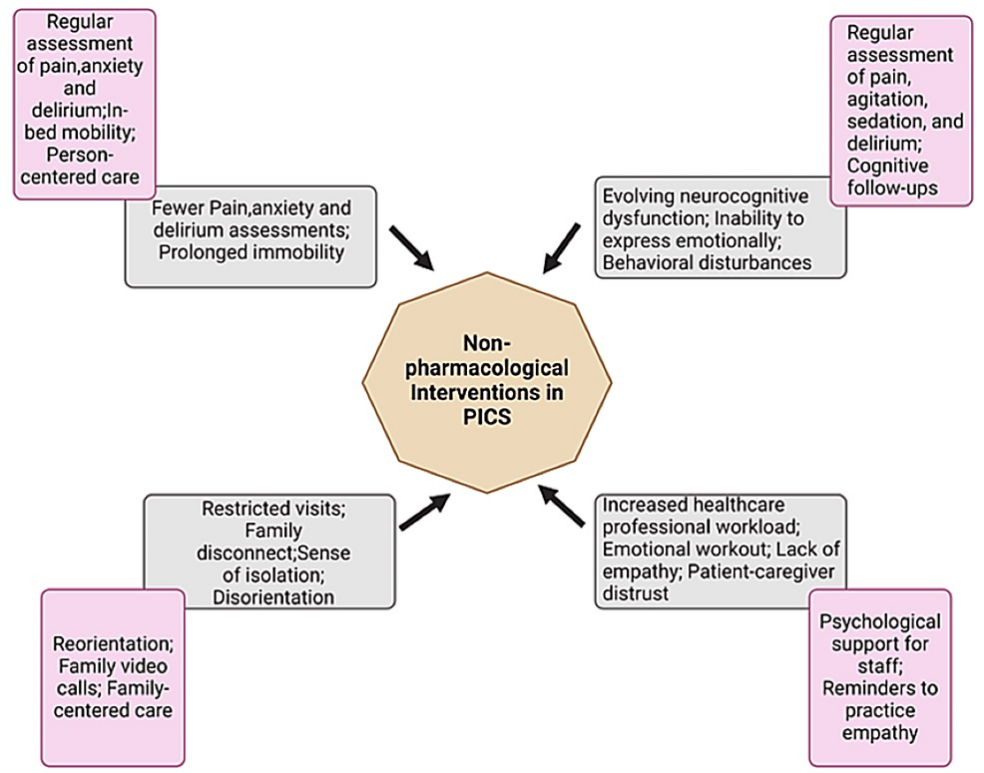
Potential factors contributing to ICU delirium and non-pharmacological interventions. Image Credits: Jean Carlo Daher

Although pharmaceutical interventions are important, it is becoming increasingly apparent that a more comprehensive approach is necessary to properly deal with the complex and multifaceted character of PICS. Nevertheless, it might be difficult to locate reliable information that substantiates the effectiveness of a multidisciplinary approach. Therefore, the importance of prevention and early detection cannot be emphasized enough [50]. While certain studies have found limited evidence on the impact of early mobilization on critically sick individuals in the ICU, subsequent research has provided new insights into the advantages of this strategy (Figure 4).

Current guidelines strongly advocate for early mobilization during the ICU stay, as research has demonstrated its beneficial impact on multiple facets of patient recuperation. These factors encompass minimizing the time spent on ventilation, reducing the duration of hospitalization, decreasing the occurrence of delirium, and enhancing muscle strength upon discharge [5,51]. Furthermore, the implementation of early mobilization has shown substantial advantages in relation to health-related quality of life and physical function. However, the effectiveness of improved exercise therapy following hospital discharge has not produced as many favorable outcomes as early mobilization [52]. Hence, commencing rehabilitation for severely ill patients within 72 hours after their admission to the ICU can improve their physical and cognitive abilities and mitigate the risk of developing PICS. Nevertheless, there is still continuous disagreement and no consensus has been achieved about the determination of the most effective rehabilitation program in the ICU setting.

In addition, intensifying rehabilitation efforts in the ICU did not yield a significant improvement in the number of days that patients were alive and were discharged from the hospital when compared to the standard level of daily rehabilitation in the ICU for individuals enduring mechanical breathing. The effectiveness of rehabilitation was greater in trials when the control group underwent a low-dose physical rehabilitation regimen of less than five days per week. This implies that active rehabilitation activities may not provide any extra advantage compared to regular everyday therapy [53]. Moreover, the key measures that have been demonstrated to effectively avoid the long-term functional deficits linked to PICS involve promoting early mobility in ICU patients, as well as implementing intensive physical and occupational therapy [3].

Psychological therapies are advantageous for critically ill patients with adaption difficulties, such as anxiety and sadness. In order to enhance results, it is imperative to administer these therapies throughout the patient's stay in the ICU and early rehabilitation stages [51]. Patients with severe psychological disturbance may require specific therapies such as trauma-focused cognitive behavioral therapy (CBT). Nevertheless, despite receiving therapy, long-term evaluations of individuals who have survived their stay in the ICU reveal a persistent and significant occurrence of psychological consequences one year following the critical illness [52]. However, CBT is widely regarded as the primary non-pharmacological treatment for the majority of psychological problems linked to PICS, such as depression, anxiety, and PTSD [50].

Family involvement in the care of ICU patients, the use of ICU diaries, and the provision of psychological support can improve psychological well-being [51]. The Society of Critical Care Medicine advocates for the inclusion of family members in the care of patients in the ICU. They also suggest the establishment of "open" ICUs, which enable families to observe the medical team's efforts and actively participate in patient care. "Open" ICUs are distinguished by their flexible visiting practices, which include flexible hours, no restrictions on the number or age of visitors, and regular daily contact with families.

Studies have demonstrated that including family members in the treatment of patients in the ICU can decrease the likelihood of experiencing depression and PTSD three and 12 months after being discharged [3,17]. Studies strongly endorse the widespread usage of ICU journals to chronologically record occurrences, incorporating contributions from both staff and family members, along with images of patients [17]. ICU journal maintenance has been discovered to mitigate symptoms of PTSD and can function as a complete instrument for delivering support and care to both the patient and their family [3].

Challenges and barriers to effective management

The intricate nature of symptoms, along with the absence of a singular ICD-10 classification, obstructs the process of identifying and implementing screening measures [54]. Furthermore, a dearth of awareness and information among providers, patients, and caregivers is a substantial obstacle. The lack of precise definitions and rules impedes the proper resolution of the problem, resulting in some clinicians being completely uninformed of the diagnosis. Patients may withhold symptoms from primary care providers due to a lack of understanding of accessible options or a fear of being misunderstood [54,55]. A study conducted at a tertiary medical facility identified a lack of information and comprehension of PICS as the primary obstacles to enhancing the quality of life for patients affected by this condition [55]. The study reported that the patients and caregivers faced challenges in recalling and comprehending their ICU encounters and post-ICU prospects, resulting in feelings of seclusion and obstacles in seeking assistance and therapy. Furthermore, healthcare practitioners had a limited understanding of patient and caregiver perspectives and faced challenges in identifying and treating PICS, which hindered their ability to enhance the quality of life for vulnerable patients.

Post-ICU results are dependent on the availability of ICU resources, the associated costs, and the procedures used. The global literature highlights common difficulties such as limited resources and elements within the healthcare system. The challenges encompass limited autonomy in decision-making, apathy towards survivors, and communication deficiencies, in addition to the financial constraints and psychological distress endured by survivors [56]. However, there are facilitators who recognize the importance of PICS, are sympathetic to the needs of survivors, and are conscientious professionals [57]. Nevertheless, the application of mitigation strategies encounters substantial obstacles, such as the inability to address risk factors effectively and frustration caused by the disparity between academic recommendations and their practical execution. Using the World Health Organization's (WHO) International Classification of Functioning, Disability, and Health (ICF), early mobilization is identified as a potential protective factor that can reduce physical disability after intensive care (PD PIC). However, only a small portion of US ICUs regularly implement early mobilization due to a lack of staff, a bad unit culture, and the perception of increased workload [58]. In Germany, general practitioners are more relied upon for treating trauma in a medical setting compared to trauma induced by other factors, such as PTSD [59]. However, the availability of psychological therapies is still restricted due to the presence of social stigma.

To comprehend post-ICU rehabilitation, it is necessary to consider patients' biopsychosocial context in a comprehensive manner. This includes elements such as debility, cognitive and emotional problems, altered role identities, and environmental influences [9,55,60]. Although the priority of saving lives is crucial, the ICU settings can be isolating, prioritizing severe medical interventions over the preservation of brain function [60]. Medications and extended sedation, which are frequently used in ICUs, might potentially impair cognitive function. Post-ICU cognitive deficits frequently remain unnoticed and/or unattended, resulting in inadequate adherence to medication and post-care requirements.

An essential strategy for effectively addressing common requirements after early discharge is to adopt a proactive, individualized, and interdisciplinary approach. It is imperative to acknowledge the hazards associated with polypharmacy, including the excessive use of particular drugs. Medication reconciliation and coordinated care are important issues that have not been adequately addressed [9,51]. Furthermore, the insufficient participation of psychiatry in the ICU underscores the necessity for enhanced psychological assistance. Nevertheless, differing interpretations of psychiatry's function and possible disagreements among healthcare professionals continue to impede a cooperative approach [61]. In order to effectively treat the physical, cognitive, and psychological concerns of individuals with PICS, it is crucial to have proactive interdisciplinary collaboration. To reduce PICS and improve post-ICU outcomes, it is important to prioritize awareness, allocate resources effectively, provide patient-centered care, and promote communication.

Future directions and research needs

PICS is a significant medical and social issue that imposes a cost on healthcare professionals, patients, their families, and healthcare systems [62,63]. The field of intensive care medicine has made considerable advancements throughout time, resulting in a rise in the utilization of ICUs. The rise in utilization has led to a corresponding increase in hospital admissions, patient survival rates, and the use of rehabilitation services. Currently, approximately 25-50% of ICU patients will develop new and worsening cognitive, physical, and psychological issues throughout their hospitalization. These consequences need to be managed in order to prevent long-term disability and post-ICU morbidity [63,64]. However, it is worth noting that up to 80% of ICU patients do survive their stay. Consequently, there is a demand for investigating and advancing novel treatments such as exercise therapy administered using virtual reality and rehabilitating robots to prevent and address PICS [10].

The objective of exercise and rehabilitation treatment for ICU patients is to enhance physical well-being, restore functionality, and avoid impairment. During and after ICU admission, this approach has demonstrated its efficacy and safety in preventing and managing PICS [10]. Nevertheless, a significant constraint occurs since ICU patients frequently exhibit an inability and/or reluctance to engage in ET due to various reasons. The integration of cutting-edge and new supplementary treatments, such as virtual reality, to enhance ET has produced favorable outcomes in effectively involving patients who might otherwise be uncooperative [10]. This delivery mode completely submerges the patient in a simulated environment that promotes active movement and has demonstrated efficacy in diminishing anxiety, delirium, and pain levels. Virtual reality has been successfully implemented in many small-scale studies, demonstrating significant patient interest and minimal adverse effects. Another growing method of delivering extraterrestrial therapy is through the use of robotic technology. This may involve the use of rehabilitation robots and/or robotic exoskeletons to assist in the process of mobilization. Nevertheless, the existing study on this topic is scarce, and further investigation is necessary to evaluate the practicality and security of these automated devices.

An important constraint in the care of PICS is the absence of patient follow-up, either by virtual means or in person. The absence of a health policy supporting PICS outpatient clinics in any healthcare system has resulted in many healthcare institutions having to allocate their own funds to pay for PICS follow-up clinics [65]. Establishing a dedicated fund for patient follow-up will greatly enhance the capacity of healthcare staff to provide adequate infrastructure, personnel, and equipment for effective PICS follow-up. Furthermore, the use of telephone or internet-based telemedicine for patient follow-up can overcome limitations in human availability and address the issue of post-ICU patients being unable to attend clinics due to limited mobility [65].

Furthermore, the active participation of family members in treatment plans, follow-up, and overall patient care has proven to be highly beneficial in the management of PICS, as reported by multiple family members. Furthermore, research has demonstrated that family participation can effectively alleviate the anxiety and PTSD symptoms experienced by family members both during and after their loved one's hospitalization [62,66]. Some of the studies included in the article have been listed in Table 1.

**Table 1:** List of the studies conducted on post-intensive care syndrome, its risk factors, and various pharmacological and non-pharmacological interventions.

S. No.	Study	Methodology	Outcomes
1.	Tejero-Aranguren et al. [4]	This study focuses on observing ICU patients hospitalized for at least a week, requiring mechanical ventilation for over three days, and experiencing delirium or shock. It investigates the prevalence and risk factors associated with post-intensive care syndrome (PICS), its impact on physical, cognitive, and mental health by conducting a univariate analysis using assessment scales (Pfeiffer, Barthel, Impact of Event Scale-6 and Hospital Depression and Anxiety Scale).	The study included 87 patients with an average APACHE II (acute physiology and chronic health evaluation) score of 16.5. These patients spent an average of 17 days in the ICU. The incidence rate of PICS was found to be 56.3%. The most significantly impacted aspect is mental health, followed by physical and cognitive health, in that order. Diverse risk factors identified for various health domains.
2.	Kim et al. [6]	The study aims to collect data on the health post-discharge of 121 ICU survivors between August 1st and December 31st, 2019, focusing on the impact of post-intensive care syndrome on long-term survivors' quality of life and measuring physical, cognitive, and mental issues.	The quality of life is negatively linked to mental, physical, and cognitive health issues, with factors such as well-being, education, medication use, and relationship status influencing this relationship.
3.	Docherty et al. [14]	A prospective cohort study to conduct a secondary analysis of multi-center data on critical illness recovery among three distinct groups.	The study highlights a strong correlation between anxiety, pain, and depression across all cohorts, emphasizing pain as a common debilitating symptom, and suggests interventions targeting these areas are beneficial.
4.	Pun et al. [19]	A multicenter cohort study involving 69 adult ICUs from 14 countries aimed to identify the frequency of delirium and coma among severely ill COVID-19 patients, identify factors increasing delirium occurrence, and determine the length of stay in the ICU, using data from a RED Cap database.	Acute brain dysfunction (delirium or coma) was more prevalent in patients with acute respiratory failure compared to those without COVID-19. Factors like mechanical ventilation, restraints, and medication administration increased the likelihood of delirium the following day. Family visits reduced the risk of delirium.
5.	Flaws et al. [27]	Secondary examination derived from Tracking Outcomes Post-Intensive Care (TOPIC) study conducted a secondary examination on 132 patients, who underwent evaluation using self-assessment instruments at six months post-ICU discharge.	A significant number of patients with short ICU stays and ventilation durations experienced PICS, highlighting a potential morbidity burden that may be overlooked in clinical practice. Of these, 40 experienced at least one impairment six months post-ICU discharge.
6.	Parker et al. [34]	Systematic review and meta analysis of the prevalence, risk factors, and prevention/treatment strategies for posttraumatic stress disorder symptoms in critical illness survivors.	After a year, 20% of critical illness survivors develop post-traumatic stress disorder, with higher rates in benzodiazepine-treated patients, those with prior psychopathology, or those with disturbing ICU memories, and studies in Europe suggest ICU diaries may reduce PTSD symptoms.
7.	Davydow et al. [37]	Systematic review to critically review data on the prevalence of depressive symptoms in general intensive care unit (ICU) survivors, risk factors for these symptoms, and their impact on health-related quality of life (HRQOL).	Depression negatively impacts health-related quality of life in ICU survivors. Further research is needed to understand risk factors, but sex, age, and severity are unlikely to increase risk. Early recognition of depressive symptoms is crucial, and collaboration between intensive care physicians and psychiatrists is essential.
8.	Seo et al. [42]	Quantitative Study. Revised the 2010 Guideline based on the 2018 “Clinical Practice Guidelines for the Prevention and Management of Pain, Sedation, Agitation/ Sedation, Delirium, Immobility, and Sleep Disruption (PADIS) in adult patients in the ICU”.	The 2021 Korean Society of Critical Critical Care Medicine (KSCCM) guideline emphasizes the importance of a multidisciplinary approach in managing sedation, pain, and delirium in critically ill ICU patients, focusing on reducing opioid use, promoting comfort, and addressing sleep factors that influence recovery.
9.	Stollings et al [47]	The aim of this study was to evaluate the impact of delirium on outcomes in critically ill patients in the intensive care unit (ICU) and assess the effectiveness of routine monitoring using validated delirium instruments. The study employed a comprehensive literature review to examine the association between delirium duration and various clinical outcomes, as well as the efficacy of interventions such as the ABCDEF (A2F) bundle in improving patient outcomes.	The study found that delirium is the most common manifestation of brain dysfunction in critically ill patients and is independently predictive of adverse outcomes such as excess mortality, prolonged length of stay, increased cost of care, and development of acquired dementia. The use of validated delirium instruments, such as the CAM-ICU and ICDSC, was recommended to accurately diagnose delirium and prevent misdiagnosis.
10.	Renner et al. [51]	The study developed a guideline for PICS therapy based on current evidence, addressing 10 research questions identified by a multidisciplinary team.	The rehabilitation therapy for PICS patients should be individualized, multidisciplinary, and interdisciplinary, with multiple assessments of physical, psychological, and cognitive functions, and evidence-based guidance for improved outcomes.
11.	Zhang et al. [56]	A qualitative study conducted semi-structured interviews with 21 healthcare workers who provided follow-up services to ICU survivors, analyzed through content analysis between August and December 2022.	This study provides insights into barriers and facilitators to post-ICU follow-up services, enabling medical personnel to better utilize resources and develop strategies to overcome limitations, offering a reference for structured and systematic follow-up in low- and middle-income countries.
12.	Zbar [57]	The study involved semi-scripted interviews with 11 healthcare team members from seven ICUs in Essex County, New Jersey, and performed a thematic analysis using open, axial, and selective coding.	Educating the target population and healthcare providers about barriers that hinder the implementation of PICS mitigation measures can potentially change the current situation.
13.	Sanftenberg et al. [59]	A multicentre randomized controlled trial from Germany (PICTURE) aims to test a brief psychological intervention, based on narrative exposure therapy, for post-traumatic stress disorder symptoms following intensive care unit treatment in the primary care setting.	The primary care setting, characterized by enduring doctor-patient relationships and accessible consultations, presents favorable conditions for implementing brief psychological interventions targeting post-intensive care unit impairments. However, to optimize this potential, structured follow-up guidelines for primary care post-ICU treatment are essential, suggesting the integration of brief interventions within a stepped-care approach.
14.	Amass et al. [66]	The study assessed the feasibility and efficacy of implementing “Family Care Rituals” (FCR) as a means of engaging family members in the care of patients admitted to the ICU with a high risk of ICU mortality on outcomes including stress related symptoms in family members.	Offering opportunities such as FCR for family members to be involved with providing care for family members in the ICU was associated with reduced symptoms of PTSD. This intervention may lessen the burden of stress related symptoms in family members of ICU patients.

## Conclusions

PICS is a growing concern in the field of critical care medicine. Patients not only experience physical, cognitive, or emotional health issues, but they also face significant financial burdens as a result of extended hospital admissions. The etiology of this condition can vary from muscle weakness caused by extended immobility to immune suppression and injury to small blood vessels, resulting in polyneuropathy. The likelihood of getting PICS is strongly correlated with the severity of the disease and the complications that arise during a patient's hospitalization, as well as any pre-existing comorbidities. Elderly individuals and women are particularly vulnerable to developing PICS. Acute respiratory distress syndrome (ARDS) and the need for extended mechanical ventilation are the most significant risk factors for the development of PICS. Recognizing the need for personalized care is crucial. Utilizing various interventions such as early mobilization and psychological therapy can alleviate post-ICU complications, ultimately improving patient outcomes and reducing healthcare costs. In order to enhance comprehension and achieve more favorable results, additional research and clinical data are required to identify novel therapeutic targets and therapies for PICS to enhance patient outcomes and facilitate early detection of PICS.
